# A Body Tracking-Based Low-Cost Solution for Monitoring Workers’ Hygiene Best Practices during Pandemics

**DOI:** 10.3390/s20216149

**Published:** 2020-10-29

**Authors:** Vito M. Manghisi, Michele Fiorentino, Antonio Boccaccio, Michele Gattullo, Giuseppe L. Cascella, Nicola Toschi, Antonio Pietroiusti, Antonio E. Uva

**Affiliations:** 1 Department of Mechanics, Mathematics, and Management, Polytechnic University of Bari, via Oraboba 4, 70125 Bari, Italy; michele.fiorentino@poliba.it (M.F.); antonio.boccaccio@poliba.it (A.B.); michele.gattullo@poliba.it (M.G.); Antonio.uva@poliba.it (A.E.U.); 2 Department of Electrical Engineering and Information Technology, Polytechnic University of Bari, via Oraboba 4, 70125 Bari, Italy; giuseppeleonardo.cascella@poliba.it; 3 Department of Biomedicine and Prevention, University of Rome ‘Tor Vergata’ Facoltà di Medicina e Chirurgia Viale Montpellier, 1, 00133 Rome, Italy; toschi@med.uniroma2.it (N.T.); pietroiu@uniroma2.it (A.P.)

**Keywords:** body tracking, azure kinect, occupational safety, safety training, hygiene best practices, pandemics containment

## Abstract

Since its beginning at the end of 2019, the pandemic spread of the severe acute respiratory syndrome coronavirus 2 (Sars-CoV-2) caused more than one million deaths in only nine months. The threat of emerging and re-emerging infectious diseases exists as an imminent threat to human health. It is essential to implement adequate hygiene best practices to break the contagion chain and enhance society preparedness for such critical scenarios and understand the relevance of each disease transmission route. As the unconscious hand–face contact gesture constitutes a potential pathway of contagion, in this paper, the authors present a prototype system based on low-cost depth sensors able to monitor in real-time the attitude towards such a habit. The system records people’s behavior to enhance their awareness by providing real-time warnings, providing for statistical reports for designing proper hygiene solutions, and better understanding the role of such route of contagion. A preliminary validation study measured an overall accuracy of 91%. A Cohen’s Kappa equal to 0.876 supports rejecting the hypothesis that such accuracy is accidental. Low-cost body tracking technologies can effectively support monitoring compliance with hygiene best practices and training people in real-time. By collecting data and analyzing them with respect to people categories and contagion statistics, it could be possible to understand the importance of this contagion pathway and identify for which people category such a behavioral attitude constitutes a significant risk.

## 1. Introduction

### The Risk of Pandemics

**Figure 1:**
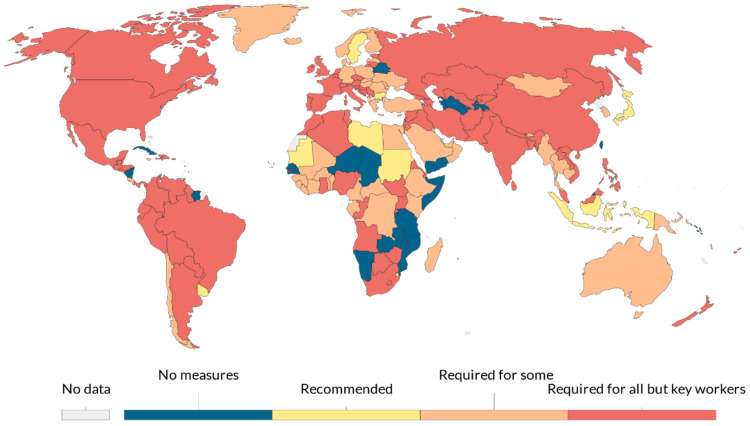
Workplace closures during the COVID-19 pandemic on 2 April 2020. There may be sub-national or regional differences in policies on workplace closures. The policy categories shown may not apply at all sub-national levels. A country is coded as “required closures” if some sub-national regions have required closures (source [2]).

Following the pandemic spread of the severe acute respiratory syndrome coronavirus 2 (Sars-CoV-2) and its related disease, COVID-19 [1], the world is experiencing an exceptional threat to public health involving, in only nine months, over 39 million contagions and costing over one million human lives. To contain the virus spread, extraordinary social-distancing measures (also known as lockdown) have been applied worldwide, involving school and workplace closures (Figure 1), travel limitations, and stay-at-home requirements. Though effective, such measures are not sustainable for an extended period and involve heavy repercussions, both social and economic [2,3]. Unfortunately, the pathogen has high transmissibility and infectivity [4,5]. The primary carrier of contagions is made up of respiratory droplets that are directly inhaled or come into contact with the oral, nasal, or periocular mucous by hand-mediated transmission [6,7]. As no specific therapeutics and vaccines are available for disease control, the COVID-19 pandemic is posing a great threat to global public health. Such a critical scenario is related to every pandemic event, and the threat of emerging and re-emerging infectious diseases exists as an imminent threat to human health and international security [8]. Consequently, it is of paramount importance to implement adequate solutions to enhance society preparedness to face the insurgence and coexist with such events.

Digital technologies are a crucial asset in the fight against pandemics. In the initial spreading phase, they are used to model viral activity and forecast the virus outbreak [9,10], to collect and access real-time data monitoring the spread of contagion [11,12,13], to boost the research for early illness diagnoses [14], virus genome sequencing, and accelerated drugs and vaccine development [15,16,17,18,19,20], and to fill the communication gap induced by the social distancing measures—the so-called “Infodemic” [21].

**Figure 2:**
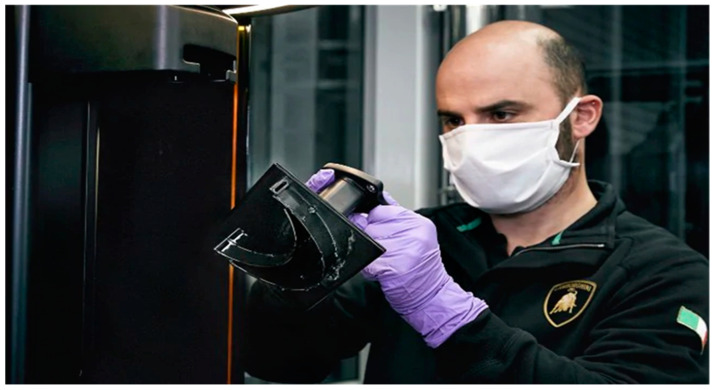
Personal Protection Equipment (PPE) used in the industrial scenario at one Lamborghini facility in Italy converted into PPE production after the COVID-19 outbreak. (Gentle permission of Automobili Lamborghini S.p.A.).

In the recovery process, innovative technological solutions have a high potential to safeguard peoples’ health by enhancing the capability to break the chain of contagion. The World Health Organization (WHO) guidelines provide specific hygiene best practices for implementation to prevent contagion [22]. Such practices provide for:

- Environment decontamination based on periodic sanitation and ventilation and the use of specific air conditioning filters;
- Use of Personal Protection Equipment (PPE) such as gloves, masks, face shields, and gowns (Figure 2);
- Strict compliance with Behavioral Protection Practices (hereinafter BPPs), both in interpersonal relationships and at the workplace. BPPs provide for: avoiding crowding conditions, maintaining at least a 1-m distance between individuals, using PPE correctly, frequently washing hands, and avoiding hand–face contacts [23].

Avoiding hand–face contacts is also essential for individuals using PPE. Wearing masks could increase hand–face contacts due to frequent moving of the mask, unconscious “fidgeting,” or skin discomfort generated in the perioral area. Furthermore, there is a need for further investigation on this topic, given the lack of a specific evaluation of the relative role of inhalation and hand-mediated transmission [6]. Novel technologies have already been used to support compliance with BPPs. For instance, Integrated Circuits industries developed solutions based on Radio Frequency Identification (RFID) or Wi-Fi networks for monitoring safety distances and overcrowding [24].

Whilst these solutions exploit body-location tracking and do not allow monitoring of hand–face contacts, another technology—the full-body tracking one—can be exploited to support compliance with BPPs. This adopts two main approaches: body-worn sensors and digital image processing.

Body-worn sensors systems use accelerometers, gyroscopes, goniometers, and magnetometers that are either held, worn, or attached to body parts. These systems are relatively inexpensive and have advantages such as a relatively quick adoption and integration with existing PPE. Furthermore, they are largely embedded in daily life-usage devices such as smartwatches or fitness smart-bracelets. Recently, D’Aurizio et al. presented the No Face-Touch system, a solution using Micro-Electro- Mechanical-Systems-based sensors embedded in typical smartwatches, able to estimate hand proximity to face and notify the user whenever a face-touch movement is detected [25]. The authors implemented two algorithms to detect face-touch movements. The first algorithm correctly detects 91.3% of the face-touch movements by measuring the magnetic field emitted by a handmade magnetic necklace and evaluating the hands’ distance. The second algorithm does not use the magnetic necklace and has a 92.6% accuracy. False positives affect the specificity of the system. The rationale for developing the second algorithm relies on some drawbacks hindering the use of this kind of sensor in the daily life scenario. Inertial-Measurement-Unit (IMU) based technologies suffer from ferromagnetic disturbances, and accelerometers require periodic registration because of the inherent drift error [26]. Besides, using PPE may hinder the effective positioning of such sensors that, if not correctly designed, can be invasive to normal activities [27,28,29].

Among digital image processing-based systems, the marker-based ones are the golden standard. These systems use either reflective or active markers attached to body parts. Their complexity ranges from single-camera to multi-camera setups enabling orientation flexibility and full 3D body-joints tracking [30]. Despite their accuracy, such solutions are limited to laboratory studies because they require a complex and expensive setup difficult to reproduce in the daily life scenario [31]. The other solutions, based on digital image processing, use either 2D Red-Green-Blue (RGB) images or depth images. The former relies on machine-learning algorithms to segment the body parts and detect their 2D space position. Several researches investigated using such technology for detecting hand over face gestures [32,33,34]. This task is considered very challenging to handle because hand occlusion is one of the main factors limiting the accuracy of facial analysis systems. As the face becomes occluded, facial features are either lost, corrupted, or erroneously detected. Nevertheless, this technological solution achieves a good tracking performance (e.g., 90% accuracy for hand–lip occlusions) [32], but the skin color, at the basis of the hand detection algorithms, can influence its effectiveness. Consequently, such a solution is not easily exploitable in environments where BPPs require wearing PPE, such as gloves and face masks.

Despite such a limitation, a web application for detecting single user’s hands-over-face gestures was presented in March 2020—the don’t touch your face [35]. The application can detect face occlusions and notify them in real-time by using Artificial Intelligence (AI) and a standard webcam. However, it is unable to identify the touched face areas and does not return any user-activity log. Furthermore, it requires a user-specific training phase and its reliability is strongly dependent on the user’s positions relative to the webcam.

Such limitations can be overcome by exploiting low-cost body tracking systems based on depth cameras (also known as RGB-D cameras). RGB-D cameras acquire a depth map of the framed scene used by body tracking systems to feed AI algorithms for identifying body parts and their 3D coordinates. These cameras were initially introduced on the consumer market as gaming interface sensors, such as the ASUS Xtion, the Orbbec3d, and the Microsoft Kinect families. As image-based technologies, they offer great flexibility thanks to the easy setup and ready-to-use functionalities. Thus, despite limited success in the gaming market, they have been used in other fields ranging from research to rehabilitation. Today they find new applications within the Industry 4.0 program. In particular, the Microsoft Kinect family has found applications in many research areas, including human–computer interaction [36,37,38], occupational health [39,40,41], physiotherapy [42,43,44,45], and daily life safety [46]. Furthermore, recently Microsoft released the Azure Kinect DK (also known as k4a) [47]. It is an evolution of the previous Microsoft RGB-D sensor, the Microsoft Kinect V2, whose accuracy has been widely studied [29,48,49,50].

This work aims at investigating whether and how commercially available low-cost RGB-D-based tracking systems can be effectively and easily exploited for monitoring people’s compliance with behavioral hygiene measures in the workplace.

In the remainder of this paper, the authors propose the HealthSHIELD tool, a software prototype based on the Azure Kinect camera. The prototype has been developed for monitoring, in real-time, people’s tendency to touch their face, increasing their awareness with real-time warnings, and providing statistical reports to limit, as far as possible, any sources of contagion from pathogens such as Sars-CoV-2. The authors describe the approach followed to define the system requirements, the functionalities, and a preliminary validation study assessing the system’s accuracy in laboratory conditions. Finally, the authors draw their conclusions and discuss the pros and cons of the proposed solution.

To the best of the authors’ knowledge, there are no other applications of RGB-D-based technologies for monitoring operator compliance with BPPs counteracting virus contagion in the workplace.

## 2. Materials and Methods

### 2.1. System Requirements

The authors identified, as an application scenario, a fixed workstation such as a position in a factory production line, an office desktop, a reception for triage in a healthcare facility, or a supermarket checkout. The authors focused on two hygiene measures to be applied in this scenario:

- Avoiding hand contacts with the perioral and periocular areas;
- Maintaining an interpersonal distance of at least 1 m.

The system requirements were defined considering the following needs:

- Monitoring people’s behavior in the application scenarios for providing proactive feedback;
- Collecting behavioral data for statistical reports;
- Studying the relative importance of direct inhalation of droplets and hand-mediated contamination for better understanding the virus contagion pathways.

This pool of needs is the rationale at the bases of the system requirements. In detail, the prototype should:

1. Be transparent to the users (that is, not hindering working activities);
2. Be easy to set up in the workplace;
3. Allow 3D body-part tracking (i.e., hands, eyes, ears, and nose);
4. Allow a reliable real-time hand–face contact detection;
5. Distinguish between hand–face contact areas;
6. Generate real-time hand–face contact warnings;
7. Monitoring the distance between people;
8. Log the hand–face contact events for further behavioral studies and eventual task-execution procedure redesign.

**Figure 3:**
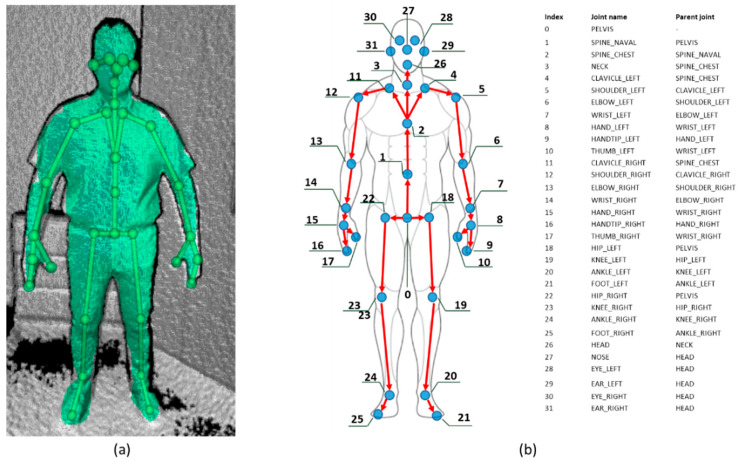
The data returned by the Microsoft Azure Kinect Body Tracking Software Development Kit. (**a**) The surface and the skeleton visualized by the Azure Kinect Body Tracking Viewer; (**b**) joint positions and respective parent joint in the joints’ hierarchy.

By mapping such requirements to the commercially available low-cost RGB-D-based tracking systems, the Microsoft Azure Kinect DK (also known as k4a) meets all of them. The RGB-D technology is transparent to the user, and the operative range of the k4a avoids any interference with normal activities. Differently from other RGB-D sensors such as the Asus Xtion and the Orbbec3d families, the k4a tracks specific body parts such as hands, eyes, ears, and nose (see Figure 3). Such tracking functionalities are ready to use. Furthermore, the k4a is easy to set up (e.g., plug and play) thanks to the availability of pre-built drivers.

Regarding the last three requirements, thanks to the availability of the Azure Kinect Sensor Software Development Kit (SDK) [51], it is possible to easily implement a software tool that fulfills them.

### 2.2. The HealthSHIELD Tool

The HealthSHIELD tool has been implemented with the C# programming language by using the Azure Kinect Sensor SDK v1.4.0 [51], the Azure Kinect Body Tracking SDK v1.0.1 [52], the C# API wrappers for the previous SDKs v1.4.0 [53], the Windows Presentation Foundation toolkit v3.5.50211 [54], and the OpenGL.Net libraries v 0.8.4 [55].

For the main application, the tool should be in a fixed place (e.g., office desktop, industry or laboratory workstation, store checkouts, school desk, and so on) where people accomplish their daily life activities. People under observation are expected to face the sensor, at a distance of about two meters, without turning their face more than 45 degrees with respect to the sensor, which is positioned at a height of about 170 cm. The operative range of the sensor avoids any interference with normal activities.

The tool operates sequentially with four modules. The first module, the data retrieval, acquires the raw data through the k4a and passes them to the gesture detection module. This module detects hand–face contact gestures, classifies them with respect to the face contact areas and labels the corresponding frame accordingly. The third module, the attitude monitor, processes the hand–face contacts, identify and labels the contact events, and feeds the Graphical User Interface (GUI) module (a description of the GUI module and its functionalities is available in Appendix A).

#### 2.2.1. The Data Retrieval Module

The data retrieval module connects to the k4a and uses it to collect both the 3D coordinates of the body joints and the infrared video stream. The k4a is based on a 12-Megapixel CMOS sensor and a 1-Megapixel time-of-flight depth camera. The field of view of the depth camera ranges from 75° × 65° to 120° × 120° with operative distances of 0.5–3.86 m and 0.25–2.21 m, respectively [47]. The body tracking algorithms of the body tracking SDK return for each frame a hierarchical skeleton composed of 32 joint objects (Figure 3). It is worth noting that the k4a can track the eye- the nose- the ear-, and the hand joints.

#### 2.2.2. The Gesture Detection Module

The Gesture Detection module uses the 3D joint coordinates to detect frames with hand–face contacts and classify the contact area using custom heuristics implemented by the authors. Such an approach allows avoiding training any machine learning algorithms.

**Figure 4:**
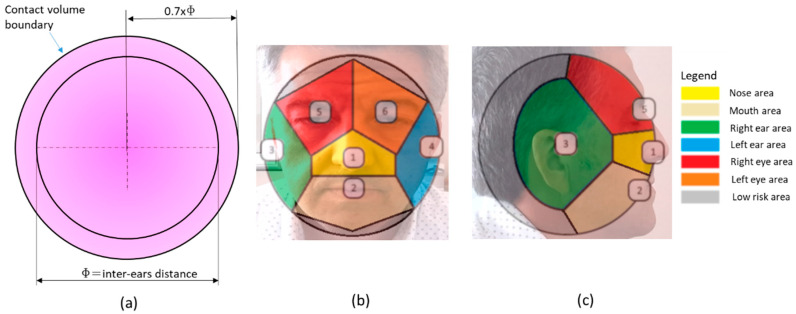
(**a**) The sphere modeling the head and the contact volume (in light violet). (**b**) A schematic of the contact areas on the head modeled as a sphere and projected onto the frontal plane; each number corresponds to a joint, in detail: (1) nose, (2) mouth, (3) right ear, (4) left ear, (5) right eye, (6) left eye. (**c**) A schematic of the contact areas on the head modeled as a sphere and projected onto the sagittal plane.

To detect the hand–face contacts, the head has been modeled as a sphere centered in the midpoint between the two ear joints and having the diameter equal to the distance between them (Figure 4a). A contact volume has been defined corresponding to a contact sphere centered as the sphere modeling the head. If a thumb joint enters the contact volume during the operator’s activity, the corresponding frame is labeled as a hand–face contact.

The contact volume definition represents a tradeoff choice. On the one hand, defining a wide contact volume would involve detecting false contacts, on the other hand, narrowing it would involve missing true contacts. The contact volume was defined by adopting a trial and error approach by iteratively increasing and decreasing the contact sphere radius to find an acceptable threshold.

The contact sphere radius has been defined as 70% of the distance between the ear joints to adapt to the operator’s anthropometrics. It is worth noting that according to the anthropometric dimensional data for a 40-year-old American male, the 50th percentile head breadth is equal to 15.7 cm, the 5th percentile is equal to 14.8 cm, and the 95th percentile is equal to 16.8 cm [56]. Consequently, between the 5th and the 95th percentile, the contact sphere radius ranges from 10.36 to 11.76 cm.

After detecting a frame with a hand–face contact, this module classifies the contact area by evaluating the face joint nearest to the thumb one. At this end, the authors implemented custom heuristics to distinguish between the mouth area, nose area, ear area, eyes area, and contacts in a low-risk area far from these regions (Figure 4b,c). By doing so, the sphere surface is divided into areas similarly to a Voronoi diagram [57].

To distinguish contacts in a low-risk area, which is far from the mouth, the nose, the eyes, and ears, the gesture detection module uses a secondary distance threshold. Each contact frame is labeled as a low-risk area if the thumb joint distance from the nearest joint exceeds the secondary distance threshold. The threshold has been defined empirically similarly to the contact volume radius, and it is equal to 75% of the distance between the two ear joints.

**Figure 5:**
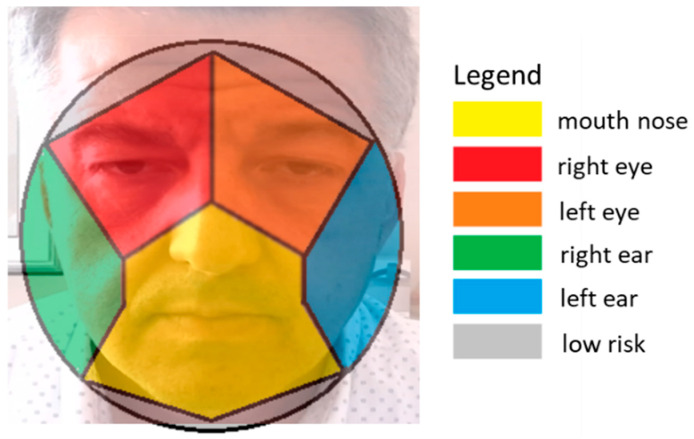
A schematic of the contact areas onto the sphere modeling the head corresponding to the six contact event classes detected by the HealthSHIELD event classifier.

At the end of the contact area classification, each contact belongs to one of the seven areas in Figure 4b,c. Finally, as the mouth area is near the nose one, these areas are merged into a single one in a subsequent processing stage. By doing so, the contact areas are reduced from seven to six (Figure 5). At the end of this stage, each frame has a contact-state label (i.e., contact or no-contact), and each contact frame has the corresponding contact area label.

#### 2.2.3. The Attitude Monitor Module

This module connects the gesture detection module to the graphical user interface one and operates in two moments of the observation—in real-time and at the end.

In real-time, the module compares each newly acquired frame with the previous one. If the contact state label changes from no-contact to contact or the contact area label changes, this module sends a signal to the interface module, indicating a contact event and the contact area. By doing so, the module groups sequences of frames with the same contact-state label as a contact event with the corresponding contact area label.

In addition, the module checks, for each frame, if there is more than one person in the viewing range and calculates the distance between the hip center-body joint of each one of them. If the interpersonal safety distance is not respected, the module sends a signal indicating an under-security distance condition to the interface. During the real-time observation, all frame labels are stored in a buffer together with their timestamps.

At the end of the observation, the module processes the data in the memory buffer, generates a statistical report on the observed worker’s attitude, sends it to the interface module, and stores it in a comma-separated values file.

**Figure 6:**
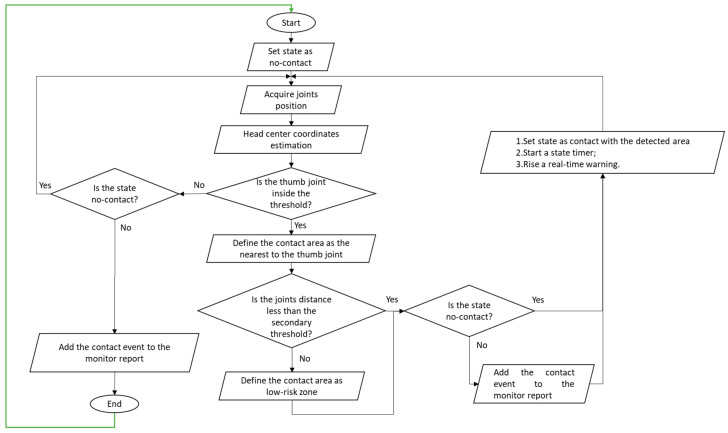
Hand–face contact detection and classification algorithms; the green arrow connecting the end node to the start one indicates that the algorithm is iterated for each newly acquired frame.

The flowchart in Figure 6 explains the algorithm on the basis of the detection and classification heuristics and the procedure used to keep track of the frames contact-state. It is executed iteratively for each newly acquired frame, as evidenced by the green arrow connecting the end node to the start one.

### 2.3. Validation Procedure

As previously described, the detection module together with the attitude monitoring module act as event classifiers by detecting and classifying the contact events into six classes—that is the mouth–nose, the right-eye, the left-eye, the right-ear, the left-ear, and the low-risk contact events (Figure 5).

Regardless, during daily life activities, it is possible to raise the hand and cause face-occlusion events without actual face contacts (i.e., gestures in which one hand is raised at the face level and occludes it to the camera field of view without actually touching the face). It is worth noticing that the HealthSHIELD classifier does not detect the occlusion events and only considers the corresponding frames as no-contact state frames.

To answer the research question, an experiment was carried out to evaluate the HealthSHIELD classifier’s capability to distinguish between the aforementioned contact event classes. At this end, the detection and classification capabilities of a human observer were used as a baseline.

After this evaluation, the collected data were further processed. The contact event classes were clustered into two main classes. The first one, named risk contacts, contains the mouth–nose, the right-eye, and the left-eye contact events. The second one, named no-risk contacts, contains the right-ear, the left-ear, and the low-risk contact events. After clustering, it was possible to evaluate the overall capability to distinguish between risk and no-risk contact events.

Two hypotheses were formulated:

**Hypothesis** **1** **(H1).** *The HealthSHIELD classifier classifies the events into the six contact areas in agreement with the human observer*.

**Hypothesis** **2** **(H2).** *The HealthSHIELD classifier classifies the events into risk and no-risk contact areas in agreement with the human observer*.

#### 2.3.1. Experimental Setup

One volunteer (male, age 49 years, height 178 cm, weight 92 kg) stood in front of the k4a sensor which was about two meters at the height of 170 cm from the ground. Simultaneously, next to the Kinect sensor and at the same height from the ground, an RGB camera framed the scene.

The experimental procedure consisted of two phases. First, the volunteer had to mimic random face-contact gestures (there were three sessions, each one lasting about 15 min). The volunteer during this phase could also execute gestures corresponding to face occlusions. During gesture execution, the k4a sensor framed the volunteer, and the HealthSHIELD tool ran the analysis on a high-end laptop with CUDA-enabled GPU. Simultaneously, the RBG camera recorded the gestures’ execution, and a specific tool recorded the HealthSHIELD GUI on the laptop screen to visually evaluate if the contact events were detected and classified in real-time. Second, an experimenter carefully analyzed the RGB recordings to detect and annotate the hand–face contact events, the execution time, and the contact area. The experimenter also detected the face-occlusion gestures.

An event-based procedure was applied to guarantee the synchronization between both recordings’ timestamps, as in [29]. At the end of this phase, it was possible to compare the events detected and classified by the HealthSHIELD tool classifier and the ground truth obtained from the experimenter.

#### 2.3.2. Data Analysis and Metrics

The data gathered from the HealthSHIELD tool and the experimenter allowed the generation of the confusion matrix (Table 1) and evaluation of the metrics for assessing the classifier’s reliability.

In the first row of a confusion matrix, there are the classes each sample belongs to, and in the first column, the classes in which each sample is classified. If a sample is correctly classified as belonging to its class, it is a True Positive (TP), if it is correctly classified as not belonging to a class, it is a True Negative (TN). Correspondingly, if a sample is wrongly classified as belonging to a class, it is a False Positive (FP), and if wrongly classified as not belonging to a class, it is a False Negative (FN). By doing so, all the correctly classified samples, TPs and TNs, lie on the main diagonal. In each row, the samples outside the diagonal elements are FPs for the class of that row. In each column, the samples outside the diagonal elements are the FNs for the class of that column. The confusion matrix had seven rows and seven columns, corresponding to the six contact events and the occlusion events. By asking the experimenter to also detect the face-occlusion events, it was possible to also take into account, in the confusion matrix, the contact events missed by the classifier.

The base metrics used to evaluate the classifier’s reliability were the TPs, the TNs, the FPs, and the FNs. These metrics allowed the evaluation of three advanced metrics assessing the reliability of a classifier: the accuracy, the precision, and the sensitivity. They are defined as follows:

Accuracy = (TPs + TNs)/(TNs + FNs + FPs + TPs) * 100(1)

Precision = TPs/(TPs + FPs) * 100(2)

Sensitivity = TPs/(TPs + FNs) * 100(3)

Furthermore, the classifier’s overall accuracy and the strength of the classification accuracy as expressed by Cohen’s Kappa were evaluated [58]. Cohen’s Kappa is a measure of agreement between the classifier and the ground truth. Cohen’s Kappa considers how much the agreement could be expected by chance.

After evaluating the classifier reliability with respect to the seven classes, its general capability to distinguish between the risk contacts, the no-risk contacts, and the face-occlusion events was evaluated. To this end, the confusion matrix for the classification into these classes was generated and the corresponding reliability metrics were assessed.

## 3. Results

**Figure 7:**
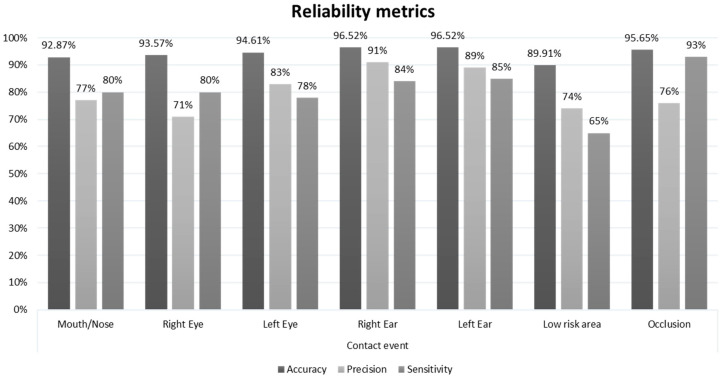
The reliability metrics assessed for each class of the seven-class classification.

By analyzing the recordings, the experimenter observed 575 hand–face gesture events. Table 1 reports the confusion matrix for the classification in seven classes. The graph in Figure 7 reports the reliability metrics.

The seven-class classification achieved an overall accuracy of 79.83%, supported by Cohen’s Kappa, which was equal to 0.764.

**Figure 8:**
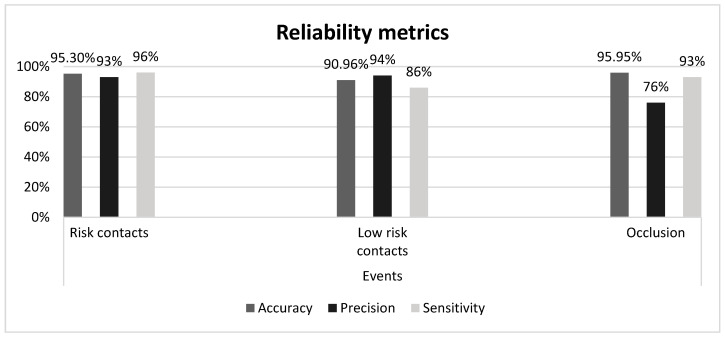
The reliability metrics assessed for each class of the three-class classification.

Table 2 reports the confusion matrix for the three-class classification. The graph in Figure 8 reports the reliability metrics.

The three-class classification achieved an overall accuracy of 90.96% supported by Cohen’s Kappa equal to 0.851.

## 4. Discussion

In the seven-class classification, the system achieves an overall accuracy of 79.83% supported by Cohen’s Kappa of 0.735, implying a substantial agreement in the Landis and Koch scale [58]. This result confirms hypothesis 1: the HealthSHIELD tool classifies the events in substantial agreement with the human observer. More importantly, hypothesis 2 is also confirmed. After clustering the contact events into the two main classes, risk and no-risk, the classification achieves 90.96% accuracy supported by Cohen’s Kappa at 0.876, corresponding to an almost perfect agreement in the Landis and Koch scale.

Regarding the system performance, a tradeoff choice in the definition of the sphere radius and the secondary distance threshold was applied to detect, as much as possible, the risk-contact events. By doing so, the performance was reduced in terms of precision for this class and in terms of sensitivity for the low-risk contact class. For the occlusion class, the authors preferred reducing, as much as possible, the FP rate at the expense of the detriment in the precision metrics.

The visual evaluation of the capability to detect and classify the events in real-time gave a positive response, not evidencing any noticeable delay between the contact timestamp and the GUI alert notification.

In an empirical comparison, the HealthSHIELD tool outperforms the automatic “don’t touch your face” web-based application. This result is due to different video technology. The web-based application uses a standard RGB camera that does not have a stereoscopic mink so that, if the face area is occluded by a hand, there is a high probability of false-positive detection. Stereoscopy-based technologies can overcome this issue, but they imply an increased cost in terms of hardware and software implementation. The proposed prototype, based on depth images, suffers much less from this problem and distinguishes between the contact areas.

Apart from better performance, the prototype has two main advantages. First, it transparently collects data, and second, the time requested to obtain such data in digital format is equal to the activities’ duration. If the same analysis were carried out offline by a human observer, it would require much more time, both for contact events’ detection/classification and data digitalization.

Another advantage consists of being ready to use not requiring any training phase or setup effort. It can be moved in an observation place with the only necessity being keeping individuals under observation inside the sensor’s field of view.

Nevertheless, the prototype has some limitations. Two main factors can negatively affect the system’s tracking accuracy. The first one consists of possible occlusions of body parts to the sensor’s field of view. The second one consists of the inability to segment body parts and track the body joints. Indeed, the segmentation process exploits the depth variations between body parts in the depth image. Sometimes, it is possible that, if the fingers are in proximity to the face, given the small variation of depth between the points of the two surfaces, the system cannot segment them. In the worst-case scenario, the system is unable to track the thumb joint. In other ones, the joint 3D-coordinates suffer from slight jittering. These limitations can cause two main issues. First, if the individual is back facing the sensor, it is impossible to correctly track the face and thumb joints. Similarly, if turning more than 45 degrees with respect to the sensor, one of the hands is likely to be occluded by other body parts and, consequently, it is not trackable. Second, when the thumb joint is near the face, the body tracking system could not track it with sufficient accuracy for the aforementioned motivations. Therefore, the monitoring system can generate two kinds of errors: it can miss that contact, or it can detect multiple successive contacts because, given the slight jittering, the thumb joint enters and exits the contact volume.

Moreover, given the lockdown condition, the preliminary test has been carried out only in a single-use case scenario. Nevertheless, as soon as the security measures’ loosening allows, the authors plan to validate the prototype extensively. They also plan to evaluate its effectiveness as a training tool that, thanks to real-time warnings, and periodic reports regarding the hand–face gesture attitude, can positively influence the compliance with BPPs by establishing a virtuous circle. The authors also plan to evaluate the user acceptance of such a warning system based on visual and acoustic feedback and compare it to a warning system based on a wrist-worn vibrating bracelet.

## 5. Conclusions

By analyzing the current practice for the containment of the Sars-CoV2 outbreak and searching for the available technological solutions, the authors designed and developed the HealthSHIELD, a software tool based on the Microsoft Kinect Azure D-RGB camera aimed at monitoring compliance with the BPPs. The preliminary study assessing the system detection effectiveness was encouraging and returned a 90.96% accuracy. The results evidence that low-cost RGB-D technologies can be effectively exploited for monitoring the compliance with some of the BPPs for virus contagion containment.

The capability to break the chain of contagion by improving people’s compliance with hygiene best practices and avoiding risk conditions (e.g., meeting people and being closer to them than the recommended security distance) will turn useful in the post-lockdown phase. Considering that the pandemic will not disappear abruptly and that successive waves are expected, the wide adoption of the solution can potentially support and secure the recovery of daily life and support the preparedness of the society to face future pandemics.

Apart from warning people in real-time and providing BPP training support, by systematically collecting data and analyzing them with respect to people’s categories and contagion statistics, it will be possible to aid the understanding of the importance of this contagion pathway and identify which people categories, such a behavioral attitude, constitutes a significant risk. In such a hypothesis, proper solutions could be applied (e.g., occupational health professionals could define how workplaces should be redesigned or the work shift job-rotation could be modified to lessen risky events [59]). Consequently, the HealthSHIELD tool could find applications in most places where people act in a shared environment (e.g., schools, public offices, factory shopfloor, hospital triage areas).

## Figures and Tables

**Table 1:** The confusion matrix for the seven-class classification.

Truth Data							
	Mouth Nose	Right Eye	Left Eye	Right Ear	Left Ear	Low Risk Area	Occlusions
**Mouth/Nose**	74	7	14	0	0	1	0
**Right Eye**	12	55	1	9	0	1	0
**Left Eye**	7	0	64	0	5	1	0
**Right Ear**	0	1	0	69	0	6	0
**Left Ear**	0	1	1	0	68	6	0
**Low risk**	0	5	2	4	7	64	5
**Occlusions**	0	0	0	0	0	20	65

**Table 2:** The confusion matrix for the three-class classification.

Truth Data									
**Classifier Results**			**Mouth Nose**	**Right Eye**	**Left Eye**	**Right Ear**	**Left Ear**	**Low Risk**	**Occlusions**
			**Risk Contacts**			**Low-Risk Contacts**			
	**Mouth/Nose**	Risk contacts	234			17			0
	**Right Eye**								
	**Left Eye**								
	**Right Ear**	Low-risk contacts	10			224			5
	**Left Ear**								
	**Low risk**								
	**Occlusions**		0			20			65

**Figure A1:**
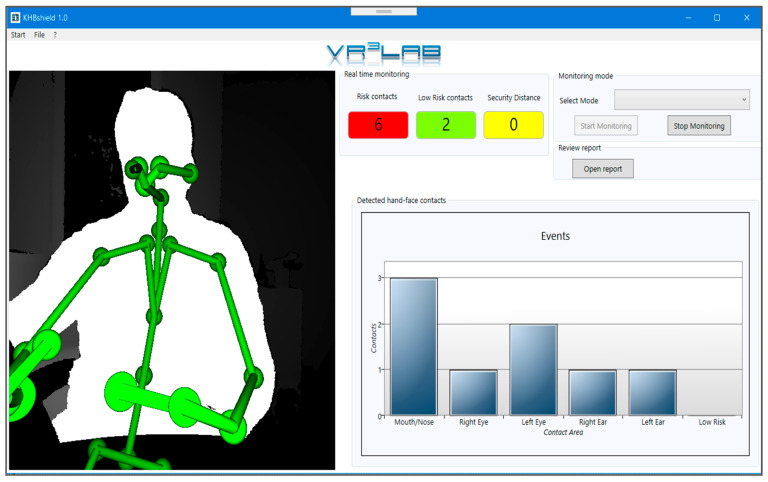
The HealthSHIELD tool’s main window for the real-time monitoring modality. In the display area, a worker at a seated workstation.

**Figure A2:**
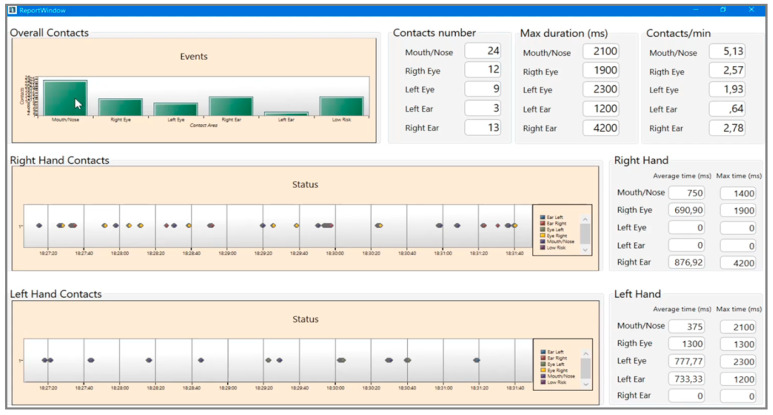
The report window of the HealthSHIELD tool prototype.

## Supplementary Materials

The following are available online at https://www.mdpi.com/1424-8220/20/21/6149/s1, Video S1: Video HealthSHIELD tool.

## Appendix A. The HealthSHIELD GUI

The GUI has two windows, the main and the report windows. The main window (Figure A1) allows the monitoring of people’s behavior. The left panel visualizes the acquired depth stream with the tracked skeleton overlapped on the worker’s body silhouette (visualized with the depth stream for privacy matters). On the upper right side, a dropdown menu allows a selection between two monitoring modalities: real-time monitoring and background monitoring.

In real-time modality, an expert can observe the worker’s behavior (please see the provided Video S1). The GUI notifies both the under-security distance and the hand–face contact events and updates, in real-time, a bar chart with the contact events’ statistics binned into the respective categories. Two counters update the total amount of events that are further grouped into two classes: contacts onto a risk region (i.e., mouth/nose and eyes areas) and contacts onto a low-risk region (i.e., ears and low-risk areas). A further counter updates the under-security distance events.

The Video S1 provided as supplementary material shows the HealthSHIELD tool working in the real-time monitoring modality.

The background modality allows people to be trained to be compliant with the BBPs (i.e., avoiding hand–face contact gestures and keeping the security distance). In this modality, if the monitored person has a monitor connected to the system, a window pops up on it and displays real-time visual and acoustic warnings at each face-contact and under-security distance event.

The report window allows a detailed analysis of each monitoring report (Figure A2). It visualizes a graph with a sequence of the contact events with the corresponding initial timestamp and the duration, a bar chart with the overall statistics, the mean contact event time-frequency in the observed period (expressed as events per minute).
